# Clinical, Epidemiological and Experimental Approaches to Assess Adverse Health Outcomes of Indoor Biomass Smoke Exposure: Conclusions from An Indo-Swedish Workshop in Mysuru, January 2020

**DOI:** 10.3390/toxics8030068

**Published:** 2020-09-05

**Authors:** Mahesh Padukudru Anand, Kjell Larsson, Gunnar Johanson, Harish C. Phuleria, P. Veeranna Ravindra, Lena Ernstgård, Ulaganathan Mabalirajan, Murali Krishna, Lena Palmberg, Krystal J. Godri Pollitt, Swapna Upadhyay, Koustav Ganguly

**Affiliations:** 1 JSS Medical College and Hospital, JSS Academy of Higher Education and Research, Department of Pulmonary Medicine, Mysuru 570015, India; 2 Unit of Integrative Toxicology, Institute of Environmental Medicine (IMM), Karolinska Institutet, SE-171 77 Stockholm, Sweden; kjell.larsson@ki.se (K.L.); gunnar.johanson@ki.se (G.J.); lena.ernstgard@gmail.com (L.E.); lena.palmberg@ki.se (L.P.); 3 Environmental Science and Engineering Department, Indian Institute of Technology Bombay Powai, Mumbai 400076, India; phuleria@iitb.ac.in; 4 CSIR—Central Food Technological Research Institute, Mysuru 570015, India; raviravindra1@gmail.com; 5 CSIR-Indian Institute of Chemical Biology, Kolkata 700032, India; um.rajan@iicb.res.in; 6 Foundation for Research and Advocacy in Mental Health, Mysuru 570015, India; muralidoc@gmail.com; 7 Faculty of Health and Social Care, Edgehill University, Lancashire L39 4QP, UK; 8 Department of Environmental Health Sciences, School of Public Health, Yale University, New Haven, CT 06510, USA; krystal.pollitt@yale.edu

**Keywords:** household air pollution, biomass, COPD, particle, smoke, lung

## Abstract

This report summarizes the outcome of a workshop held in Mysuru, India in January 2020 addressing the adverse health effects of exposure to biomass smoke (BMS). The aim of the workshop was to identify uncertainties and gaps in knowledge and possible methods to address them in the Mysuru study on Determinants of Health in Rural Adults (MUDHRA) cohort. Specific aims were to discuss the possibility to improve and introduce new screening methods for exposure and effect, logistic limitations and other potential obstacles, and plausible strategies to overcome these in future studies. Field visits were included in the workshop prior to discussing these issues. The workshop concluded that multi-disciplinary approaches to perform: (a) indoor and personalized exposure assessment; (b) clinical and epidemiological field studies among children, adolescents, and adults; (c) controlled exposure experiments using physiologically relevant in vitro and in vivo models to understand molecular patho-mechanisms are warranted to dissect BMS-induced adverse health effects. It was perceived that assessment of dietary exposure (like phytochemical index) may serve as an important indicator for understanding potential protective mechanisms. Well trained field teams and close collaboration with the participating hospital were identified as the key requirements to successfully carry out the study objectives.

## 1. Introduction

Exposure to biomass smoke (BMS) is considered a risk factor for the development of chronic lung diseases like chronic obstructive pulmonary disease (COPD) [1,2,3]. About one billion people worldwide do not have access to electricity and nearly three billion people (41% of global population), particularly in the rural communities of low and low-middle income countries (LICs and LMICs), use biomass fuel and traditional stoves for heating and cooking purposes [4]. Globally, there are 1.3 billion tobacco smokers [5]. Thus, BMS exposure is considered a global risk factor for the development of COPD and other chronic lung diseases in the same order of magnitude as tobacco smoking. However, the adverse health outcomes of BMS exposure remains an overlooked global public health research topic.

Biomass fuel includes wood, charcoal, twigs, grass, agricultural crop residues, and dried animal dung [6]. BMS contains respirable particulate matter (nanoparticles, PM_1.0_, PM_2.5,_ PM_10_), carbon monoxide (CO), carbon dioxide (CO_2_), oxides of nitrogen (NOx) and sulfur (SO_2_), polycyclic aromatic hydrocarbons (PAHs), free radicals, aldehydes, volatile organic compounds (VOCs), and chlorinated organic compounds [6]. Almost 100 times higher PM_10_ levels (20,000 µg/m^3^) have been recorded in households using biomass fuel with poor ventilation compared to the limits set by the World Health Organization (WHO) and the Environmental Protection Agency (EPA) [6,7,8]. 

The use of biomass fuel is closely linked to gender inequality [1]. Women and adolescent girls are exposed for the longest duration because they spend more time in proximity to the biomass smoke while cooking for 4–6 h daily in poorly ventilated dwellings [1,6,7]. Children also get exposed from birth as they stay with their mothers while cooking [1]. Therefore, exposure to BMS starts in utero, and continues during the peak phase of alveolar development (birth–9 years old), adolescence (10–19 years; period of peak lung function attainment) and continues into adulthood. In the Ugandan FRESH AIR study, Frederik van Gemert and colleagues (2015) [9] reported BMS exposure since childhood as the cause of early onset of COPD (30–40 years) in 40% of people. COPD most often occurs in people more than 40 years of age who smoke or have done so earlier in life with an average of 20 pack-years [10]. Interestingly, even though 95% of Indian households now have access to liquified petroleum gas via national schemes, it is not the primary choice of fuel among the poor sector of the society due to the ease of access to freely available biomass and reliance on uncertain and irregular income sources [11]. We apprehend that this trend will unfortunately be even more dominant during the ongoing Coronavirus disease 2019 (COVID-19) pandemic and the period following it due to the devastating socio-economic effects involved. Therefore, studies on BMS exposure-mediated chronic health effects are important to successfully implement the goal 3.9 of the United Nation’s “Agenda 2030” aimed to address healthy lives and promote the well-being of all at all ages, and to reduce the number of deaths and illness from air, water, and soil pollution and contamination-related diseases [12].

The Mysuru study on Determinants of Health in Rural Adults (MUDHRA) [13] previously reported that a minimum threshold of biomass exposure index (BMEI) of 60 is necessary to have a significant risk of developing chronic bronchitis in women [14]. Chronic bronchitis is the most common comorbid condition associated with COPD. BMEI is calculated based on the average number of hours spent cooking daily multiplied by the total number of years spent in cooking [13]. Recent studies suggest that the pathophysiology of tobacco smoke-induced COPD and BMS induced COPD to be different. BMS-induced COPD exhibits disproportionately greater bronchial involvement, less emphysema, more frequent hypoxia, and increased gas trappings compared to tobacco smoke-induced COPD [15,16,17,18]. These observations suggest that the phenotype of COPD may be related to specific prototypes of environmental/extrinsic exposures, which in turn implicate exposure-specific molecular patho-mechanisms. This also indicates that BMS-induced adverse respiratory health effects may also be distinct. This concept is also supported by our recent findings showing a distinct systemic chemokine-cytokine signature among tobacco smoke-induced COPD and BMS-induced COPD subjects [19]. Furthermore, we reported [19] significantly increased serum concentrations of chemokines C-C motif ligand 15 (CCL15), CCL27, and C-X-C motif chemokine ligand 13 (CXCL13) among biomass smoke-exposed subjects without chronic obstructive pulmonary disease compared to biomass smoke-exposed subjects with COPD with comparable exposure. CCL27 and CXCL13 are homeostatic chemokines whereas the inflammatory CCL15 or leukotactin-1 is a chemoattractant of T-cells and monocytes [19,20]. These findings further underline the plausibility of exposure-specific effects and the fine balance of chemokine and cytokines during the inflammatory process, immune cell trafficking and homeostasis to be critical between health and disease states [19,20]. 

To brainstorm the various current and future research activities for assessing BMS-exposure-associated adverse health effects in the MUDHRA cohort [13,21,22], a workshop was organized at Mysuru, India in January 2020. The workshop was organized along three major themes: (a) assessment of exposure; (b) assessment of adverse health outcomes; and (c) experimental studies under controlled exposure. The workshop was held to identify gaps in knowledge and possible methods to address the identified issues in the MUDHRA cohort. Low birth weight, respiratory infections, hindered lung development, chronic lung diseases, cardiovascular-disease, endocrine disruption leading to metabolic disorders, and cognitive defects were considered as some of the most prominent and/or most plausible adverse health outcomes of BMS exposure in the MUDHRA cohort. The overarching aim of the workshop was to discuss uncertainties and to identify plausible strategies to address the abovementioned themes into future MUDHRA cohort studies. The expected outcomes were an identification of the logistics for incorporating new screening methods, potential obstacles to be overcome in the process, and assessing the overall feasibility of any proposed future data collection. This workshop summary may serve as a “statement of policy” for the ongoing and future MUDHRA projects and may also be utilized in other projects with similar settings.

## 2. The Workshop

**Figure 1:**
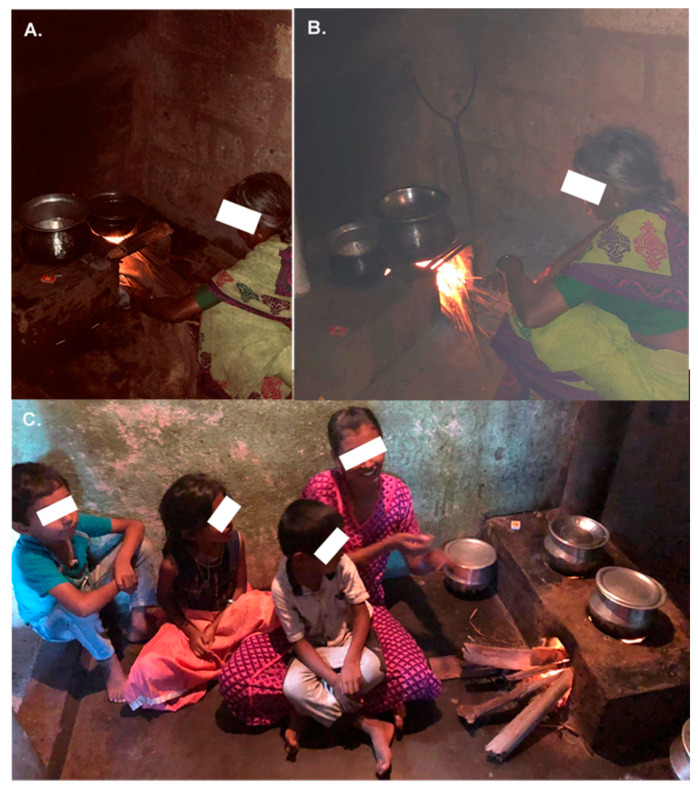
Representative pictures from field studies in Mysuru, Karnataka, India showing biomass smoke exposure among women and children during cooking. (**A**) At the start of cooking when the air is relatively cleaner. (**B**) 15 min after the start of cooking and this remains for at least 1 h after cooking. Usual cooking time is about 1–1.5 h. (**C**) Children along with mother are exposed to biomass smoke during cooking.

The scientific workshop including field visits (Figure 1) involving all the coauthors in conjunction with the MUDHRA cohort studies. The chairs (Mahesh PA and Koustav Ganguly) invited the 12 panelists to the workshop based on their expertise covering the clinical, epidemiological, experimental, and exposure assessment approaches to study air pollution-related chronic health effects with a focus on BMS exposure. The topics of the workshop were selected based on the input from the chairs and panelists. All had presented on their respective topic at the workshop highlighting the recent advances, critical gaps, and research needed in the field. The panelists were subsequently requested to submit short sections of their respective presentations. This workshop report summarizes the various clinical, epidemiological, experimental, and exposure assessment approaches to understand BMS exposure-mediated adverse health outcome in the MUDHRA cohort, work that has been completed to date, difficulties faced and the work planned for the future, which can be applied in other settings as well.

## 3. Themes

In this section each of the three major themes are discussed along with a summary of the feasibility analysis and strategies discussed during the workshop to accomplish them in ongoing and future MUDHRA studies.

### 3.1. Assessment of Exposure

It is well known that airborne pollutants such as cigarette smoke and BMS may cause COPD and other respiratory diseases. However, smoke from combustion processes is a complex mixture and the composition varies depending on various factors (e.g., the material burnt and its moisture content, the wind speed/oxygen supply, the temperature of the fire and the aging of the smoke/distance from the fire). Moreover, little is known about the relation between BMS composition (and concentration of smoke components) and health outcomes (dose–response). Therefore, well-designed prospective longitudinal field studies with simultaneous measurements of exposure and health effects under realistic conditions are highly needed. Various approaches for personal exposure monitoring, spot sampling, to continuously logging low-cost microsensors suitable for large-scale filed studies are needed. Focus on exposures of particular concern such as particulate matter (fine and ultrafine size fractions), and gas pollutants (i.e., aldehydes, CO, total VOCs) are warranted. Lastly, active observation and notes taking during field studies is extremely important for identifying anomalous observations and to reduce exposure misclassification. Many of the components of BMS may cause or contribute to various lung-related adverse health effects via different mechanisms, e.g., CO causing hypoxia and impaired mucociliary function, acrolein, and other aldehydes causing irritation and PAHs causing cancer. Aldehydes and other reactive compounds may adsorb smoke particles, thereby significantly altering the toxicity of the latter. Moreover, the concentration of inhaled smoke components, smoke, and dosimetry will vary depending on the distance to the fire, the wind speed and direction (outdoors), and air ventilation (indoors). There are limited data related to the composition of BMS, and the dose–response relationship of BMS exposures including individual components and adverse health outcomes. Exposure duration (measured as BMEI) is the widely used parameter to assess indoor cooking time at present. 

Exposure to airborne pollutants: As the exposure to BMS varies considerably both temporally and spatially, continuous sampling is preferable to short-term and spot sampling, and personal sampling is preferable to stationary sampling. Furthermore, it is crucial that the personal sampling is carried out close to the breathing zone (i.e., within 30 cm from the nose), as the BMS concentrations are likely to vary markedly even within short distances, e.g., during cooking over an open fire. With respect to continuous monitoring, passive (diffusive) samplers are a good choice for the sampling of VOCs. The advantage of passive sampling, as opposed to active, pump-based sampling, is that the devices are small and lightweight, and hence subject compliance is higher. Moreover, they can be purchased at a relatively low cost and can easily be positioned near the breathing zone and thus can be used for longer term exposure monitoring without additional efforts. All these aspects are advantageous in large-scale field studies. There are several passive samplers on the market, one attractive example is the “Fresh Air Wristband”, which contains a commercially available triethanolamine-coated pad to collect NO_2_ and a polydimethylsiloxane sorbent bar to sample VOC and PAH [23]. Other passive NO_2_ and VOC samplers include small badges and tubes impregnated with a certain solvent or adsorbent [24,25]. These samplers have been extensively used for assessing long-term (1–2 weeks to annually averaged) exposure to gaseous pollutants in urban cohort studies [26,27,28,29]. However, the use of these passive samplers in rural households to capture BMS exposures is still limited [30]. Such passive sampling devices combined with gas chromatography-mass spectrometry, and UV-visible spectrophotometry makes it possible to detect, identify, and quantify a variety of substances. The concentration in air can be back-calculated from the amount on the adsorbent, the sampling duration and the sampling rate. The latter, in turn, depends on the geometric constant of the sampler and the diffusion coefficients of the compounds of interest. For these calculations it is important to assure that the sampler does not become saturated, especially when exposure levels are difficult to anticipate.

A limitation with passive samplers is that only average concentrations of each compound are obtained. To overcome this limitation, passive sampling may be combined with the use of direct reading and logging instruments. This would help identify each subject’s variability in exposure and, in particular, exposure peaks which may be of major importance for health effects. The logging instrument does not necessarily have to capture all or even many BMS components. Several devices are available, based on different detector principles. For example, CO and CO_2_ may be measured based on infrared spectrometry or electrochemical detection, VOC based on photoionization detection, and particles (number, mass concentration, and size distribution) based on, e.g., laser diffraction or dynamic light scattering [31,32,33]. These logging detectors are available in many sizes price classes, from large and impractical to very small and easy to carry, and from relatively well documented high-end to low-cost devices. Unfortunately, the latter ones are often more or less “black boxes” with respect to, e.g., detection principle, cross-reactivity, resolution, detection limit, calibration principle (if claimed), and response time.

Whatever device is chosen, it is obviously essential to check their performance and stability prior to initiating large-scale studies. Problems that may emerge include technical failure, short battery life, interference by humidity and other (irrelevant) compounds (cross reactivity), baseline drift, sensitivity drop, cross reactivity with other (irrelevant) compounds, and slow response. Care should be taken to calibrate the instrument at regular intervals to assure a zero baseline, that it reflects the true concentration and to account for possible cross reactivity.

The simple low-cost portable instrument that can record PM_1_, PM_2.5_, PM_5_, and PM_10_ along with total VOC, CO_2_, and formaldehyde concentrations over a period of time is easy to use. Additionally, temperature, relative humidity, and CO levels are also feasible to measure. This is mainly because of the simplicity of the instrument and the fact that field workers do not need to engage much for the data recordings other than ensuring that the instruments are placed as close as possible to the breathing zone of the subjects during cooking. These are battery-operated instruments and therefore can be easily used in rural areas, where an availability of electricity is not continuous. When it comes to personal exposure assessment, the “Fresh Air Wristband” to sample mixtures of airborne contaminants is an alternative solution [23]. These samplers should preferably be worn near the collar bone (e.g., by using a magnet) to capture pollutants near the breathing zone. However, the challenge is in the analysis of samples that require sophisticated instrumentation and expertise. The sorbent bars need to be carefully collected without contamination, carried back from the field using cold packs, and stored at −20°C until further analysis. Often, the sorbent bars must be transported to a laboratory far away, which is challenging, and the analysis is expensive. For the MUDHRA cohort, we aim to further optimize the use of a “Fresh Air Wristband” in the field to capture pollutants in the breathing zone, necessary logistics for transportation, and analysis. In the near future we plan to utilize them on a larger scale.

Dietary exposure or phytochemical index: The phytochemical index can be calculated as the percentage of the daily energy derived from phytochemical-rich foods (fruits and vegetables and prepared foods derived from these, legumes, whole grains, seeds, nuts, fruit or vegetable juices, olive oil, soy sources, wine, and beer and cider) divided by the total daily caloric intake (PI = (phytochemical kJ/total kJ) × 100) [34,35]. Evidence from epidemiological, in vivo, in vitro, and clinical trial data indicates that the phytochemicals are abundant in our regular diet and that they can reduce the risk of chronic diseases like cardiovascular disease, hypertension, diabetes etc., via anti-inflammatory, anti-oxidative, and/or anti-apoptotic effects [36,37,38]. Oxidative stress and inflammatory reactions are regarded as the primary adverse outcome pathways in the pathogenesis of chronic lung diseases like COPD as well as cardiovascular diseases. Phytochemicals with anti-oxidative and/or anti-inflammatory effects (protective effect) are easily available and thereby hold the potential to be used as nutritional supplements for the prevention and/or reduction of several chronic diseases including lung disease [39] in a cost-effective way. Although the phytochemical index provides only a rough approximation of the quantity or quality of phytochemical nutrition, it nonetheless could aid in exploring the health consequences of diets high in phytochemical-rich plant foods in the case of a BMS-exposed population. Assessing the phytochemical index [40,41] in the regular diet of a BMS-exposed population, which may be endemic to a particular region, may provide an important insight into plausible protective mechanisms. However, studies on the protective effects of phytochemicals on chronic lung diseases like COPD remains largely unexplored. Therefore, a dietary questionnaire needs to be included in the field studies. 

A diet recall questionnaire with specific questions on the consumption of vegetables, spices, fruits, and legumes are included during field studies. At the time of writing this report, data from nearly 500 individuals had been collected and were being analyzed for calculating the phytochemical index. It is advantageous to have in the field team a member with a background in nutrition and a good understanding of local diet as we had in the MUDHRA cohort. It is also important to include a clinical nutritionist with a good understanding of the local diet.

### 3.2. Assessment of Adverse Health Outcomes

Respiratory effects: Nearly 3 billion people are at risk of health effects due to long-term BMS exposure. Screening for adverse chronic respiratory health outcomes due to BMS exposure can be performed in the field using a validated and structured questionnaire [21] (general characteristics, demography, premorbid conditions, BMS exposure, health status), a measurement of blood oxygen saturation, exhaled CO, fractional exhaled NO, and pulmonary function (forced expiratory volume in 1 s (FEV_1_), forced vital capacity (FVC), flow volume curves) among adolescents and women. Lower than 80% FVC with a normal/high FEV_1_/FVC ratio (>0.7) may serve as an indication for restrictive lung function in the absence of a measurement for total lung volume in field studies whereas an FEV_1_/FVC ratio <0.7 will reflect COPD according to Global Initiative for Obstructive Lung Disease (GOLD) Criteria for COPD. Field screenings can be coupled with an analysis of: (i) sputum or bronchoalveolar lavage (BAL) (eosinophil, neutrophil, tuberculosis etc.); (ii) blood (differential cell count, C reactive protein, carboxy hemoglobin, oxidative stress etc.); and (iii) inflammatory, homeostatic, and dual action chemokine/cytokine signature in sputum/bronchoalveolar lavage and blood at least in well characterized subsets of subjects. It would be worth following up the lung architecture of the well characterized subsets of subjects by high resolution computed tomography.

Respiratory infections: Studies have shown that BMS-exposed children have a nearly two-fold higher risk of Acute Lower Respiratory Tract Infections (ALRTI) compared to children who are not exposed to biomass smoke. One of the most common causes of death in children is ALRTI and it accounts for nearly half a million cases in children under 5 years of age and 39.1 million Disability-Adjusted Life Years (DALY) lost annually [42]. Younger children are at a higher risk of death than older children. Among adults, according to the global burden of disease study, household air pollutions, predominantly through biomass exposure, is responsible for 2.9 million deaths annually and 81.1 million lost DALYs [42]. Twenty six percent of these deaths are due to lower respiratory tract infections [42]. 

Biomass smoke exposure increases the risk of both viral and bacterial infections and present as acute bronchitis, bronchiolitis, or pneumonia. Viral infections could be more severe in BMS-exposed subjects and could be mediated via an increase in interleukin (IL)-8 via p38 MAPK pathway [43]. Studies have also confirmed an increased risk of bacterial infections such as *Hemophilus influenzae* in subjects with BMS exposure [44]. Platelet-activating factor receptor (PAFR), and intercellular adhesion molecule 1(ICAM-1) are the binding receptors for *Hemophilus influenzae* and biomass exposure increases inflammatory cytokines such as IL-8 and PAFR, as well as ICAM-1 expression [44,45]. Phosphorylcholine is an outer surface protein produced by bacteria such as *Streptococcus pneumoniae*, *Hemophilus influenzae*, and *Pseudomonas aeruginosa*. These bacteria enter the airway epithelial cells via the receptor PAFR [45]. Though direct evidence is lacking, it is suspected that a BMS-induced increase in PAFR could increase the risk of these bacterial infections. Biomass smoke is also associated with a greater than three-fold risk of developing tuberculosis after adjusting for various confounders such as exposure to tobacco and contact with a subject with tuberculosis among others [46]. Therefore, assessment of the occurrence of respiratory infections, particularly among children, is needed. This may impact the lung development, which may in turn have an effect on the attainment of peak lung function by late adolescence/early adulthood, and eventually result in a predisposition to chronic lung diseases.

Under five mortality: One of the most important causes of under-5 mortality remains the Lower Respiratory Tract infections, the risk of which is increased on exposure to indoor BMS. Children exposed to BMS have twice the risk of a non-exposed child of developing pneumonia and more than 3.5 times the risk of developing an acute respiratory infection [47]. The Global Burden of Disease study observed that reductions in household air pollution due to the burning of solid fuels is one the most important factors associated with lower under-5 mortality. A 8.4% (95% CI 6.8–9.2%) reduction in under-5 mortality in the world between the years 1990–2017 has been attributed to a reduction in the use of biomass fuels [42,47,48,49]. Meta-analyses observed an increased risk of pneumonia in children exposed to fossil fuels. But providing improved stoves and chimneys did not significantly reduce mortality in children, suggesting that shifting to cleaner fuels may be the only option to reduce under-5 mortality due to household air pollution from solid fuels [48,49,50,51]. Hence, accounting for under-5 mortality among biomass fuel users will be an important aspect in any study population.

Field team and logistics: One of the most important requirements for successfully carrying out field screening for studies such as those in the MUDHRA cohort is to establish multiple well-trained field teams (2 members per team) depending on the targeted study size. It is important to note that these field studies are primarily carried out in rural areas that may require several hours of travel from the urban area (Mysuru city in case of the MUDHRA cohort). The socio-cultural compatibility and competence in local language is of high importance. Equal gender distribution of the field team is another important aspect (i.e., 1 male and 1 female field worker per team). It is important to have a good network with the respective village administration and communities to perform the screening in a seamless way. Training of field workers includes the different aspects of the study objectives. The MUDHRA cohort till now has primarily focused on respiratory diseases and therefore the field teams are well trained in this aspect. In the case of the MUDHRA cohort, we have trained field teams who can complete the questionnaire (printed format), perform lung function measurements using a hand-held spirometer according to the American Thoracic Society/European Respiratory Society guidelines as well as FeNO and eCO measurements. It takes about 2.5–3 h per subject (adolescent or adult) to complete the measurements. We have recently included the measurement of the indoor air pollution level using a simple low-cost portable instrument that can record PM_1_, PM_2.5_, PM_5_, and PM_10_ along with total VOC, CO_2_, and formaldehyde concentration over a period of time. Additionally, temperature, relative humidity, and CO levels are also measured. One critical improvement suggested during the workshop is the digitalization of the questionnaire in the field. This will increase the efficiency to a good extent. On the other hand, one needs to consider that the internet connection in rural areas may not be always optimal, and thus a back-up with a printed questionnaire booklet is important along with the taking of field notes. Biomass burning for cooking takes place during odd hours (early morning or early evening) in these families. Thus, the field team needs to plan before-hand their travel plans after communicating with the respective families in order to measure indoor air pollution levels. Hence, significant micro-level planning and coordination is required. 

It is feasible to collect blood in the field when a trained nurse/technician accompanies the field team and the samples are carried back to the pathological/clinical laboratory as per the established procedure. For some of the MUDHRA projects, we have tested this logistic successfully. Moreover, this is a commonly practiced process in clinical laboratories in India. However, now we aim to collect both blood and BAL/induced sputum from a subset of the study population that has been calculated based on a power calculation to obtain informative data. Performing BAL is an invasive procedure and needs a visit to the hospital and observation, which may be challenging in this study setting with poor subject acceptance for such invasive procedures. Instead, following discussions in the workshop, we now aim to perform sputum induction. It appears feasible to bring in the subjects (in batches) from the rural areas to the associated hospital with facilities for blood testing, sputum induction, and high-resolution computer tomography (for subjects with respiratory impairment e.g., COPD). For the MUDHRA study, JSS Medical College and Hospital, a tertiary care university teaching hospital, with all the available facilities is the participating institution. Regarding the subjects, arrangements for their transport, compensation for the loss of their daily wage on top of the study participation fee has to be ensured with due permission from the institutional ethical committee. All costs for analysis and examination has to be borne by the project. As part of ethical mitigation, the study physician/s will provide consultation to the study subjects at no additional cost, if needed.

At this point in time we are carrying out studies on both adolescents (16–19 years) and adults in the MUDHRA cohort. All these procedures have been successfully established among adolescents (16–19 years) and adults, however we have not yet tested in the field younger subjects aged below 15 years. Generally, school-going children (6 years and above) can follow instructions for spirometry and for those below 6 years of age, a validated questionnaire for parents is the main resource. The team has earlier participated in the Global Asthma Network (GAN) and EuroPrevall-INCO surveys. During the screening for children, we plan to perform a validated structured questionnaire-based survey (adapted from The International Study of Asthma and Allergies in Childhood study and translated into the local language (Kannada)) followed by pulmonary function testing (FEV_1_, FVC, flow volume curves) using handheld spirometry and/ or impulse oscillometry. It may also be feasible to measure FeNO and exhaled CO in children over 6 years of age. Pilot studies in children are in the planning phase.

Cardiovascular effects: Chronic exposure to air pollutants (e.g., PM, VOCs, PAHs) over months to years has been associated with vascular dysfunction and is considered a risk factor for the development of atherosclerosis events in the long-term [52,53]. Hence, carotid ultrasound for assessing carotid intima-media thickness (CIMT), pulse wave velocity, and the augmentation index for measuring arterial stiffness, can be used to identify at-risk individuals (late adolescents and adults) for cardiovascular diseases [52,53] in connection with BMS exposure. 

Screening for a plausible predisposition to cardiovascular disease requires hospital care for at least half a day to measure CIMT, pulse wave velocity, and arterial stiffness. Thus, logistics similar to those mentioned above for blood and sputum induction are necessary for all subjects. Therefore, this would require a more intense logistic arrangement, but for the MUDHRA cohort, it seems to be feasible to organize screening for 10–15 subjects a day. A close coordination with the cardiology and radiology departments is necessary. For the MUDHRA study, cardiovascular screening, JSS Medical College and Hospital with all the available facilities is the participating institute. We would require a validation of the available questionnaire for cardiovascular screening and pilot studies are planned for this purpose.

Endocrine disruption and metabolic disorder: Several components of the BMS (e.g., PAHs, VOCs etc.) are known/potential endocrine-disruptive chemicals [54]. Long-term exposure to these chemicals may result in the disruption of the normal functioning of the endocrine system which may in turn result in metabolic disorders. Assessment of metabolic disorders therefore needs to be considered by screening the BMS-exposed population and by evaluating various endocrine disorders. Endocrine disruptors act at various levels of the endocrine pathway. They may affect the synthesis or secretion of hormones, may affect binding and conjugation to its carrier proteins, may alter the metabolism of hormones, or may modify the receptor levels and affect the binding of the hormone to its receptors [54]. PAHs released during the burning of biomass fuels can occur both in the gaseous phase as well as in the particulate matter when they are large with more than 5 rings. The key endocrine system affected by the PAHs is the estrogen pathway [54]. While hydroxylated PAHs are estrogen agonists, dioxins are estrogen antagonists. Some common clinical effects include deleterious variations in pubertal development, premature ovarian failure, irregular menstruation, polycystic ovary, fibroids, and endometriosis. PAHs disrupt key hormonal regulators such as the luteinizing hormone, the follicle-stimulating hormone, the gonadotrophin-releasing hormone, and estrogen synthesis [54]. Male reproductive systems are affected as well. Semi-volatile organic compounds and PM have been shown to reduce sperm quality and lower testosterone levels [54]. Phthalates have also been found in burning biomass fuels and is associated with testicular dysgenesis syndrome [54]. PM has also been associated with diabetes and pre-diabetes, though further studies are needed on the clinical effects of biomass-related PM [54]. 

Systemic blood markers: A broad range of systemic profiling that may include blood cell differentials, high sensitivity C-reactive protein, thyroid profile (thyroid-stimulating hormone (TSH), triiodothyronine (T3), total thyroxine (T4)), lipid profile (high-density lipoprotein, low-density lipoprotein, very-low-density lipoproteins, triglycerides etc.), cardiac markers (lipoprotein A, apolipoprotein A1, B etc.), liver profile (alanine transaminase, aspartate transaminase, alkaline phosphatase, albumin and total protein, bilirubin, gamma-glutamyl transferase, L-lactate dehydrogenase, prothrombin time etc.) can be measured for preliminary screening as well as for longitudinal follow up. Detailed blood tests can be performed based on specific project objectives. Understanding the signature of inflammatory, homeostatic, and dual action chemokine/cytokines in the blood will further help in our understanding of BMS exposure-mediated effects and their role in disease susceptibility/resistance. 

As a part of the normal screening procedure, we measure body weight and height to calculate body mass index, and hip and waist circumference ratios are also measured. Portable instruments can be used to record sleep patterns in the future, especially for cognition studies. Blood can be collected in the field, along with a trained nurse/technician, stored as per protocol, and transported back to the clinical laboratory to measure a range of systemic blood markers as mentioned above. The challenge in this case is to organize with the clinical laboratory to avail the trained nurse/technician in the field during the screening process. This may require an outsourcing of the analysis to a competent private clinical laboratory certified by the National Accreditation Board for Testing and Calibration Laboratories, India when large-scale sampling is required. This is feasible for the MUDHRA cohort study. We would require to validate the available questionnaire for metabolic screening in the MUDHRA cohort and pilot studies are planned for this purpose.

Cognitive effects: There is limited and inconsistent evidence, mainly from high income countries, indicating that growth restriction in utero may lead to lower cognition and a higher risk of depression in later life, either through impaired brain development or adverse metabolic programming. Exposure to high levels of CO since childhood due to BMS exposure may directly influence the brain development or through its effects on lung function and other potential mediations of cardiometabolic risk factors [55]. This highlights the importance of conducting studies with a life course approach to better understand the relationship between exposures in early life, through to mid-life and late-life concerning cognitive development and decline.

Cognitive function and depression can be measured using a culturally adapted and validated 10/66 battery of cognitive tests and the Geriatric Mental State examination respectively [40]. These are well validated not only for use in India but across other LMICs. A normative range of scores across different cognitive domains for men and women stratified by age and attained educational levels are available. It is desirable to interview a reliable informant for evidence of any decline in cognitive function and resulting functional impairment of the participants. In studies related to examining the relationship of environmental exposures (e.g., BMS exposure), it is desirable to conduct other contemporaneous assessments of sociodemographic factors (like socioeconomic position—both individuals and neighborhoods, social mobility, cohesion, networks, and relationships) and lifestyle factors (like physical activity, use of alcohol, smoking, and dietary factors); blood tests (for glucose tolerance, insulin resistance, diabetes, dyslipidemia, anemia, vitamin B12 and folate deficiency, and hyperhomocysteinemia), physical health assessments (for hypertension, coronary heart disease, lung disorders, and stroke) and a genetic assay (e.g., for ApoE lipoprotein). These additional assessments will allow researchers to examine the mediating and/or confounding or interacting effects of a range of social, cardiometabolic, lung, genetic and life factors on the BMS exposure and cognitive function/decline by employing advanced statistical analyses like Structural Equation Modeling and Pathway Analysis [41].

This is, in our experience, by far the most challenging screening with the human resources available to us. A series of questions for a preliminary screening for cognitive functions is incorporated in the questionnaire. The field team has undergone training with experts to carry out the procedure. We have screened nearly 300 subjects as a pilot study in the MUDHRA cohort and the results are currently being evaluated by experts. The same subjects have also undergone spirometry and a detailed questionnaire for respiratory involvement and cognition assessment via the Montreal Cognitive Assessment Test and 10/66 cognitive battery test. Based on the work load of field assistants, it was decided to develop dedicated field teams to perform cognitive assessments.

It is important to examine cognitive function by the administration of culturally appropriate and education fair battery of cognitive tests were developed and validated by the 10/66 research group for the study settings. These assessments must be conducted by trained research assistants by members of the 10/66 research group locally. These tests broadly evaluate global cognition, verbal fluency, verbal and non-verbal memory, semantic memory, and visuo-spatial abilities. The MUDHRA team has extensive experience in ensuring that these assessments are conducted according to a standardized protocol, in privacy both in the community and in clinical settings. Normative values stratified by age, gender, and education levels are available for each of the four cognitive domains that are measured, allowing for easy comparisons. 

Only those who consent will undertake these assessments, but when an individual is unable to consent due to severity or the nature of cognitive problems, or communication difficulties for example following a stroke, assent/consent from the nearest relative will be sought, which will be witnessed. If participants are distressed or appear fatigued during the tests, the assessments will be discontinued. Participants are informed that their participation is fully voluntary and withdrawal from the study will not impact on their ongoing treatment and care at the hospital, or in participation in future studies. If participants are recognized as having dementia or other mental health problems, they will be referred to the Department of Psychiatry at JSS Hospital for further evaluation and treatment, at no additional expenses to the participants as part of the ethical mitigation strategy of the project. 

### 3.3. Controlled Exposure Experiments

Physiologically relevant multicellular bronchial (normal and chronic bronchitis-like) [56] and alveolar mucosa models [57] developed at the air–liquid interface can be used to understand the molecular patho-mechanism of BMS exposure following controlled exposure (acute and repeated) experiments. This would enable realistic exposure experiments in contrast to sub-cultured experiments. The majority of in vitro studies to assess pulmonary cytotoxicity and the protective effect of phytochemicals have been performed using lung cells (cell lines and primary cells) cultured under submerged conditions. Stimulants (particles, gases) are typically added directly into the cell culture media [58] and such experimental set-ups do not reflect realistic cell–cell communication and cell–particle interactions [58] apart from dosimetry-related issues. Advanced models such as those that involve adding immunocompetent cells (macrophages) on the epithelial cell surface of bronchial [59] and alveolar models can be achieved to mimic the airway physiology. Furthermore, realistic in vitro exposure scenarios can be mimicked using such models [59,60,61,62]. The physiologically relevant in vitro lung mucosal models cultured at the air–liquid interface using human primary bronchial epithelial cells (bronchial model) and representative human type II alveolar cells (alveolar model) will provide the possibility to study immunomodulatory phenomenon such as chemokine–cytokine release (apical medium representative of bronchoalveolar lavage and basal medium representative of spill over to the systemic level), extracellular matrix degradation (matrix metalloproteinases and tissue inhibitors of metalloproteinases), oxidative stress, and macrophage polarization. Furthermore, chronic bronchitis-like in vitro bronchial mucosa models may also be used to understand the phenomenon in the predisposed condition. This will allow a direct comparison of molecular outcomes assessed in human studies coupled with a detailed understanding of in vitro molecular toxicology.

Phytochemicals present in the regular diet of the targeted population can therefore also be assessed for their protective effects (e.g., anti-oxidative, anti-inflammatory) using these physiologically relevant exposure set ups [39]. Similarly, animal models (e.g., inbred mouse strains, transgenic/knockout mouse models) can also be used to study BMS-exposure-mediated mechanisms of adverse health effects (developmental, respiratory, cardiovascular, cognitive, metabolic etc.) following controlled exposure conditions (acute and repeated). Nutritional supplementation of phytochemicals with plausible protective effects can also be assessed in animal experiments. It is important to note that animal experiments may only be performed in a focused manner and not for screening purposes to keep animal usage at a minimum and follow the “3R-reduce, refine, and replace” principle [63].

While mimicking the exposure scenario in the laboratory for performing in vitro and in vivo exposures, the composition of biomass varies tremendously from real life. The biomass fuel used in real life depends on vegetation and several other factors. In contrast, in the laboratory we mainly use clean wood chips. However, in that case it is essential to perform a chemical composition analysis of the wood smoke generated in the laboratory, like the measurement of particle number concentration and size distribution, and the CO concentration of the exposure chamber so as to be within realistic levels compared to scenarios during indoor biomass fuel burning. It is extremely important to be aware of the composition of smoke to which the in vitro and in vivo models are exposed. To achieve this it was decided to assess the PAHs and VOCs of the wood smoke using polydimethylsiloxane sorbent bars used in “Fresh Air Wristbands” [23]. 

## 4. Conclusions

**Figure 2:**
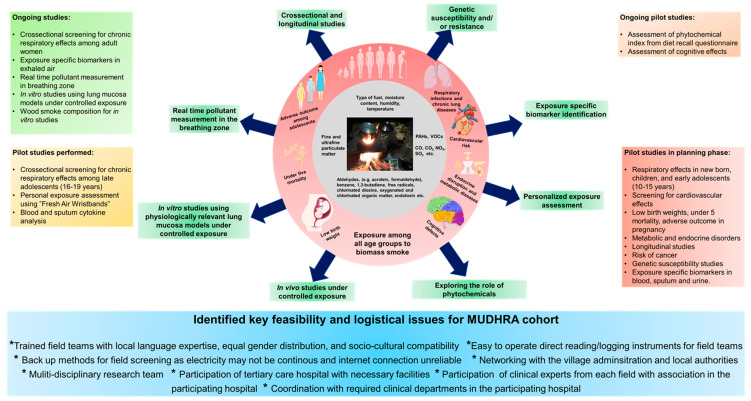
Summary of the main conclusions of the workshop.

Adverse health effects due to long-term BMS exposure is multifaceted and is not only limited to respiratory effects, even though lungs are the primary exposure route. Personalized exposure assessment (using low-cost real-time and passive samplers) in the breathing zone is an important aspect to be considered. Longitudinal cohort studies with children, adolescents, and adults are warranted to capture the BMS exposure-related adverse effects early in life so as to implement preventive measures. Controlled exposure studies can provide a significant insight regarding the molecular patho-mechanism and into the efficacy of candidate protective molecules like phytochemicals present in a regular diet. At the time of writing this report, we have nearly completed field screening of nearly 500 subjects for assessing biomass smoke-induced adverse respiratory health effects in the current ongoing study. Previous studies included screening, with the Burden of Obstructive Lung Disease questionnaire, nearly 9000 subjects, acceptable spirometry satisfying American Thoracic Society criteria for 1085 subjects, vitamin D assessments, and cognition assessments in a sub-group. The workshop identified the building-up of technically and socio-culturally competent field teams to be a key requirement for successfully carrying out the screenings at remote areas such as those required for the MUDHRA cohort. Close coordination with the participating hospitals and an involvement of study physicians associated with the participating hospital from each study domain is essential. Good networking with village administration and local authorities creates an easy access. A summary of conclusions is provided in Figure 2. The participation of experts from multiple disciplines is critical to dissect the adverse health effects of complex issues impacting on a vast section of the global population such as biomass smoke exposure.
